# It Is Time for a Multidisciplinary Rehabilitation Approach: A Scoping Review on Stomatognathic Diseases in Neurological Disorders

**DOI:** 10.3390/jcm12103528

**Published:** 2023-05-17

**Authors:** Angela Militi, Mirjam Bonanno, Rocco Salvatore Calabrò

**Affiliations:** 1 Department of Biomedical and Dental Sciences and Morphological and Functional Imaging, University of Messina, 98125 Messina, Italy; amiliti@unime.it; 2 IRCCS Centro Neurolesi “Bonino-Pulejo”, Via Palermo, SS 113, C. Da Casazza, 98123 Messina, Italy; roccos.calabro@irccsme.it

**Keywords:** stomatognathic disease, temporomandibular disorders, neurological patients, neurorehabilitation, multidisciplinary approach

## Abstract

Patients affected by neurological disorders can develop stomatognathic diseases (SD) related to decreased bite force and quality of mastication, bruxism, severe clicking and other temporomandibular disorders (TMD), which deeply affect patients’ swallowing, masticatory and phonation functions and, therefore, their quality of life. The diagnosis is commonly based on medical history and physical examination, paying attention to the temporomandibular joint (TMJ) range of movements, jaw sounds and mandibular lateral deviation. Diagnostic tools such as computed tomography and magnetic resonance imaging are used instead in case of equivocal findings in the anamnesis and physical evaluation. However, stomatognathic and temporomandibular functional training has not been commonly adopted in hospital settings as part of formal neurorehabilitation. This review is aimed at describing the most frequent pathophysiological patterns of SD and TMD in patients affected by neurological disorders and their rehabilitative approach, giving some clinical suggestions about their conservative treatment. We have searched and reviewed evidence published in PubMed, Google Scholar, Scopus and Cochrane Library between 2010 and 2023. After a thorough screening, we have selected ten studies referring to pathophysiological patterns of SD/TMD and the conservative rehabilitative approach in neurological disorders. Given this, the current literature is still poor and unclear about the administration of these kinds of complementary and rehabilitative approaches in neurological patients suffering from SD and/or TMD.

## 1. Introduction

**Figure 1:**
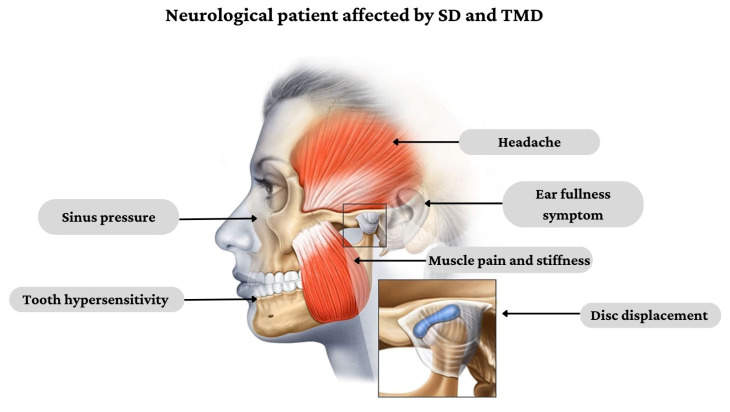
Clinical presentation of SD and TMD in people affected by neurological disorders.

The stomatognathic system (SS) is defined as a functional complex including craniofacial structures with musculoskeletal and ligamentous components, the temporomandibular joint (TMJ), oral cavity, neck and masticatory muscles [1]. Patients affected by neurological disorders can develop stomatognathic diseases (SD) related to decreased bite force and quality of mastication, bruxism, severe clicking and other TMJ disorders (TMD), which deeply affect the patients’ quality of life [2]. In fact, the integrity of SS is fundamental in activating the neuromuscular chain that initiates the swallow reflex. On the other hand, SD can also cause myofascial pain that may irradiate in different regions, such as dental arches, ears, temples, forehead, occiput, cervical spine and shoulders [3] (Figure 1), resembling atypical headaches and facial pain.

Despite the presence of SD in neurological patients, functional training for SS and TMJ has not been commonly adopted in hospital settings as part of formal neurorehabilitation. Generally, the non-pharmacological treatment for SD and TMD aims to decrease pain, induce muscle release and stabilize muscle function and joint mobility through physical therapy (PT) and/or manual techniques (MT) [4]. Among the different PT modalities, electrophysical tools (ultrasound, LASER, TENS, interferential current) have analgesic and anti-inflammatory effects, as well as therapeutic exercise alone or in combination with MT. The latter consists of the administration of hands-on techniques to improve mobility and reduce pain in the cervical spine and its upper levels [5]. In this context, osteopathic manipulative treatment (OMT), which uses both direct and indirect MT, can be easily adapted to any type of patient [6]. The OMT can be useful in people complaining of orofacial pain as it induces muscle and fascial relaxation, promoting the release of endogenous opioids due to therapeutic touch [7,8]. Moreover, reticular formation and the brainstem are strictly involved in the coordination of the masticatory cycle, which is also controlled by a central pattern generator (CPG) which, in turn, influences gait rhythmicity too. In this way, the presence of SD and TMD is not only associated with pain or functional alterations, but it also involves the central nervous system, with a documented reduction of the grey matter volume in the somatosensory cortex and the premotor cortex [9,10]. Indeed, SS and TMJ dysfunction can be related to brain and muscle/joint alterations, so a multidisciplinary approach is required to deal with these complex problems. In current clinical practice, physiotherapists usually focus more on gait recovery and arm mobility, neglecting the role of TMJ in affecting posture and gait [11]. Patients affected by TMD could manifest a depressed head posture due to forces created by masticatory muscles. In this way, dysfunctional changes in the mandibular position, influenced by proprioceptive afferents, can have an impact on gait and balance stability through muscle connections provided by cervical muscles (i.e., sternocleidomastoid muscle, elevator scapulae), dorsal muscles (i.e., trapezius), lumbar spine (i.e., intrinsic spine muscles) and pelvic girdle [12,13,14]. Since this issue is often overlooked by professional figures of a neurorehabilitation team, our review is aimed to provide more awareness about this topic, describing the most frequent pathophysiological patterns of SD and TMD in patients affected by neurological disorders and their rehabilitative approach, and to give some clinical advice about their conservative interventions.

## 2. Methods

### 2.1. Search Strategy

The review was carried out by searching on PubMed, Google Scholar, Scopus and Cochrane Library using a combination of keywords that we have reported in Table 1.

### 2.2. PICO Evaluation

Search terms were defined according to the PICO model (population, intervention, comparison and outcome) [15]. The population includes patients affected by neurological disorders such as Parkinson’s disease, multiple sclerosis, spinocerebellar ataxia, oromandibular dystonia and stroke, and complaining about SD and/or TMD. Intervention included conservative and complementary therapies, such as physiotherapy, manual treatments, OMT and splint therapy. The comparison was referred to the absence of treatment and/or the administration of just one type of conservative treatment (as shown in Table 1). Lastly, the outcome included any improvements in pain perception and TMJ/SS function shown by the patients.

### 2.3. Inclusion and Exclusion Criteria

Then, the inclusion criteria were (i) neurological patients affected by SD/TMD; (ii) pain topic; (iii) English language; and (iv) publication in a peer-reviewed journal. We excluded articles that described theoretical models, methodological approaches, basic technical descriptions as well as animal studies and conference proceedings.

### 2.4. Literature Selection

**Figure 2:**
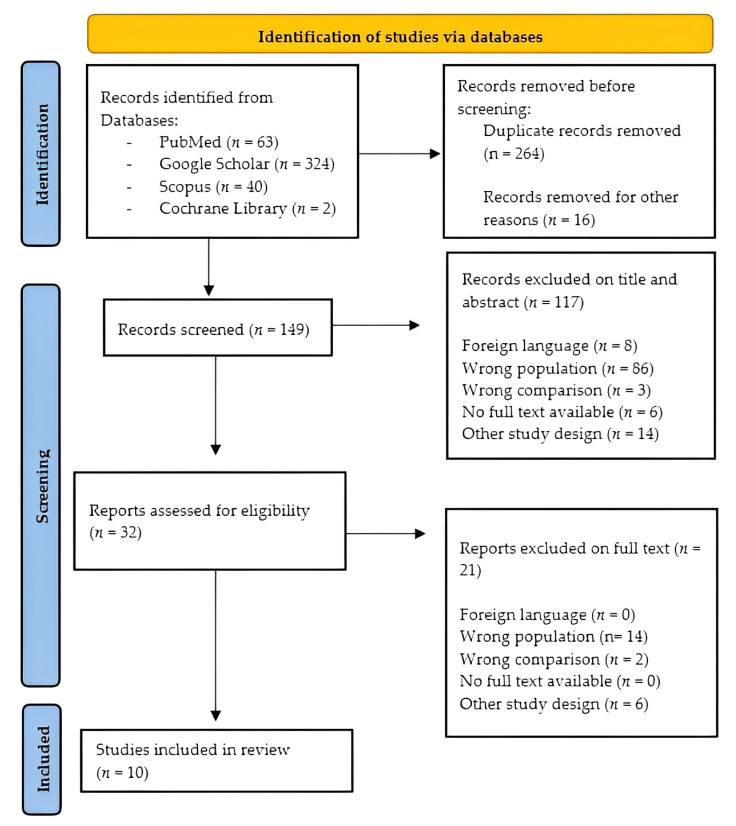
PRISMA flowchart for study selection.

We assessed the most relevant pilot studies, randomized controlled trials and case-control studies published between 2010 and 2023. Four hundred and twenty-nine articles were evaluated independently by two reviewers (MB and AM) according to title, abstract, text and scientific validity. The agreement assessment was performed using Cohen’s kappa coefficient [16]. In case of disagreement, an independent reviewer (RSC) mediated to achieve consensus. After removing duplicates (*n* = 264), 149 papers were initially screened, and only 32 were found eligible for a full assessment. Finally, only 10 articles fulfilled the inclusion criteria, as reported in the new PRISMA flowchart (Figure 2) [17].

### 2.5. Study Risk of Bias Assessment

The risk of bias in controlled studies was assessed through a revised Cochrane risk of bias (RoB 2) [18], while cross-sectional and case-control studies were evaluated through Newcastle–Ottawa scale (NOS). Specifically, the risk of bias assessment was performed by two authors (A.M. and M.B.) without the use of automation tools.

## 3. Results

We analyzed the 10 selected pieces of evidence dealing with the presence and treatment of SD/TMD in patients affected by neurological disorders. The agreement of the judge’s decision for the inclusion of the studies was Cohen’s k = 0.82, which means a perfect agreement. In particular, we classified the studies according to the association between neurological disorders and TMD and its rehabilitation treatment, reporting a summary of results in a tabular form (see Table 2).

The risk of bias assessment of randomized controlled trials, cross-sectional studies and case-control studies was evaluated, respectively, with RoB 2 (Table 3) and NOS (Table 4).

The general quality of the included studies ranged from low [19,20,21,22,24,25,27,28] to moderate [27,29]. It should be considered that the low quality of the selected evidence is likely related to the great heterogeneity among studies for the methodologies, diagnostic tools and rehabilitation treatment administered. Additionally, we excluded one study from the risk of bias assessment [19], since it is a case report.

## 4. Pathophysiology of Stomatognathic and Temporomandibular Joint Disorders in Neurological Disorders

The etiology of SD and TMD is linked to a wide range of functional, psychological and environmental factors, especially in neurological disorders in which the underlying pathology is complex. People affected by multiple sclerosis (MS) are more susceptible to developing TMD disorders [30,31]. Indeed, the concomitant presence of psychological disturbances (i.e., anxiety, depression, behavioral alteration) can exacerbate TMD disorders, as confirmed by a systematic review [30]. In this vein, it has been recently reported that patients who suffer from psychological distress are less responsive to conventional treatments for TMD, requiring a longer duration of therapy [32]. MS patients can manifest three common orofacial alterations: facial palsy, trigeminal neuralgia and/or paresthesia. However, Costa, C. et al. [19] described an unusual pattern of SD in a MS patient, which included tooth hypersensitivity, hyposalivation associated with caries, halitosis and bruxism. The latter tends to increase when occlusion is impaired, also contributing to head and neck pain. In this context, some studies hypothesized that the augmented mobility of cranial bones due to reduced bone mass density, especially in the temporal ones, expands and contracts during bruxism, increasing intracranial pressure, which can favor brain damage [20]. Another mechanism that can be involved in the pathogenesis of TMD or SD in MS patients is cerebellar dysfunction. In fact, cerebellar plaques and proprioceptive changes may lead to an increased propensity to fatigue of TMJ structures in addition to a lack of coordination of mandibular movements [30,33]. In a similar way, SD is present in patients with spinocerebellar ataxia (SCA), who often present dysarthric speech and swallowing difficulties. According to Ferreira et al. [21], SCA subjects showed a decreased bite force and hypotrophy of the masseter and temporalis muscles, with an augmented electromyographic activity. The underlying hypothesis for these electromyographic changes can be related to an increase in the amplitude and duration of motor unit action potentials, in addition to reduced muscle recruitment [34]. Moreover, the lack of coordination, especially during lateral mandibular movements in SCA patients, can be explained by the pathological alteration in cerebellum pathways, affecting the synchrony and precision of movements [35]. Furthermore, patients affected by Parkinson’s disease (PD) can manifest SD and TMD due to the presence of rigidity. Body muscle rigidity could also affect masticatory muscles in association with augmented muscle tone during sleep [22]. This condition could favor the repetitive jaw muscle activity and grinding of the teeth, named bruxism, which is considered a factor for developing TMD. Bruxism can occur both during sleep (sleep bruxism) and wakefulness (awake bruxism) [36], and its pathogenesis seems to be related to central nervous structure alterations. In fact, some antidepressant drugs, such as Selective Serotonin Reuptake Inhibitors (SSRI), can cause bruxism as a side-effect of inhibiting dopaminergic neurons [23]. This could explain why bruxism is frequent in PD patients due to the reduction of dopamine presence in basal ganglia [29]. TMD and SD were also found in other movement disorders, including dystonia. The term “dystonia” refers to prolonged or intermittent muscle contractions, causing repetitive and abnormal movements and/or postures [37]. In this context, oromandibular dystonia (OMD) is often misdiagnosed due to shared clinical features with TMD [24]. In fact, OMD is associated with masticatory disturbances such as limited mouth opening, orofacial pain and TMJ dislocations that can simulate an isolated TMD, overlooking the real etiology of the disturbance or pain. Today, six subtypes of OMD are recognized: jaw closing (i), opening (ii), deviation (iii), protrusion (iv), lingual (v) and lip (vi) dystonia. In particular, the jaw-closing subtype is related to a loss of reciprocal muscle inhibition that greatly limits mouth opening, especially during speaking or eating, worsening the patient’s quality of life. In addition, dystonia tends to expand to other muscles, including orbicularis oculi, neck and shoulder muscles [38]. The pathophysiology of SD in OMD patients is still unclear, although some studies found that functional movement disorders, as well as dystonia, present a hypoactivation of the supplementary motor area and abnormal connectivity of those brain areas designed to select or inhibit movements [39]. The onset of SD and TMD in stroke patients depends on the extent and site of the vascular lesion, which can affect cortical areas, or motor-neuron pools of cranial nerves in the brain stem, causing sensorimotor deficits in SS. The presence of facial and masticatory muscle dysfunctions has been demonstrated in post-stroke patients, including weakness and hypotonus of the masseter, orbicularis oris, mylohyoid and digastric with an increase of thickness in these muscles [40]. In detail, the most common TMD in post-stroke patients seems to be related to disc displacement, which alters the structural relationship with condyle, thus producing a click sound when the mouth opens due to translation movements. This alteration could be chronic as it interferes with the simple opening of the mouth during speech or eating, and the disc becomes progressively more dislocated. Another hypothesis is that forward head posture, due to inefficacy to maintain postural alignment, causes an overload in posterior cervical muscles that can influence the TMJ by changing the position of the mandibular condyle and, consequentially, its functioning [25,41,42].

## 5. Diagnostic Methods and Tools for TMD

**Figure 3:**
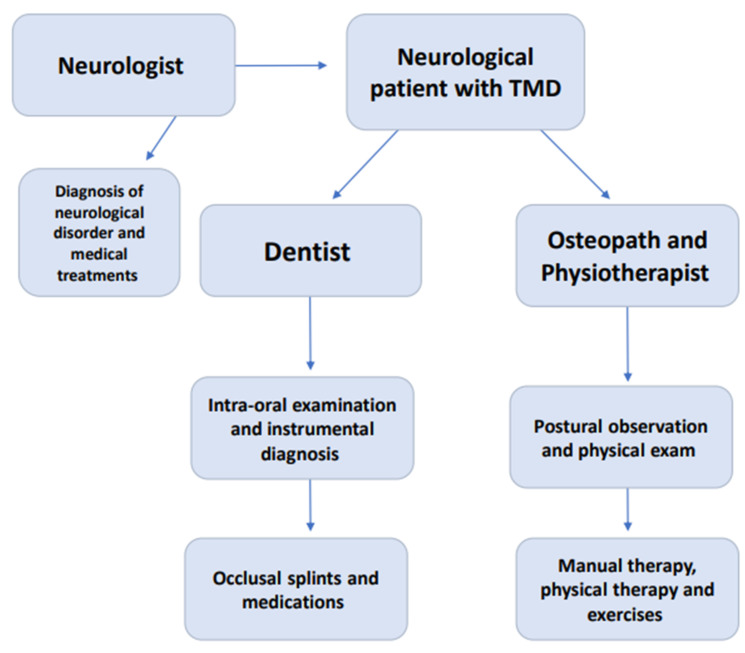
Theoretical diagnostic and therapeutic paradigm for neurological patients affected by TMD.

When orofacial pain occurs, patients commonly consult dentists or gnathologists, although osteopaths or physiotherapists can primarily identify a TMD during a physical examination and manual treatment [43]. In fact, the diagnosis of TMD is based on medical history and physical evaluation findings (Figure 3).

An in-depth anamnesis is the first fundamental step to guide the clinician to carry out the most relevant physical examination and diagnosis. A comprehensive history for a patient with orofacial pain includes the main complaint, medical history, dental history and psychosocial history. The patient should provide the dentist with a full description of the symptoms and the reason why the patient is seeking care. In this context, clinicians should investigate the quality of pain (i.e., burning, stabbing and blocking pain), which movements or activity exacerbates the pain and how long it has been present. In addition, clinicians should ask patients to indicate their pain point(s) with their fingers, figuring out the pain localization [44]. To more objectively rate the pain, different scales can be used in current clinical practice, such as the McGill Pain Questionnaire (MPQ), for the multidimensional detailed evaluation of orofacial pain. However, it takes a lot of time and requires good patient compliance. As an alternative, the short form of the MPQ (SF-MPQ) can be easily administered since it measures the pain intensity through the scores from the Present Pain Intensity (PPI) and the Visual Analogical Scale (VAS), also including sensory and affective scores that form the MPQ descriptors [44,45]. Otherwise, unidimensional pain scales are widely used in clinical evaluation routines and include VAS, the Verbal Rating Scale (VRS), which contains a list of adjectives marked up with a number describing five different levels of pain intensity; the Numerical Rating Scale (NRS), which ranges from 0 (no pain) to 10 (worst possible pain); and the Face Pain Scale (FPS), which uses seven emoticons to describe the pain sensation and is particularly useful in those patients with reduced compliance and/or aphasia [45]. As an adjunct, the Helkimo Clinical Dysfunction Index (HCDI) is a quick and simple test for the specific evaluation of TMD, assessing limitations in mandibular movements, joint function and also pain [46]. For research purposes, the neurophysiological methods, including laser-evoked potentials (LEPs), could be reliable assessment tools for painful syndromes, including TMD. According to de Tommaso [47], LEPs in trigeminal neuralgia and TMD present a smaller amplitude than healthy controls, suggesting trigeminal nociceptive system dysfunctions and neuropathic pain.

Furthermore, clinicians should observe the patient’s head and neck alignment with the whole body, hemifacial asymmetry, paying attention to abnormal mandibular movements, decreased joint range of motion and jaw sounds (i.e., clicking, popping, crepitus and grating), which can be related to anterior disc displacement (i.e., the click is produced during mouth opening) or to recapture the displaced disc (i.e., a second click is heard during mouth closing) [48]. In particular, the maximum mouth opening (MMO) is determined by measuring the interincisal distance, which is considered restricted when it is inferior to 35 mm. Restriction in MMO from 25 to 30 mm can be caused by intracapsular problems, such as disc displacement blocking the translation of the condyle. In this condition, the clinician describes the end-feel, which identifies the characteristics of TMJ restriction, as “hard”. Otherwise, a restricted mouth opening of 8 to 10 mm associated with a “soft” end-feel is most certainly of muscle origin. During MMO, two types of alteration can occur: deviations and deflections [47]. A deviation refers to any shift of the jaw midline that disappears during continued opening movement. Generally, it is caused by disc displacement with a reduction in one or both TMJs. A deflection consists of any shift of the midline to one side that becomes great during opening and does not return to the midline, and it reflects a restriction in one joint [49,50]. Indeed, lateral jaw movements should be about 12 mm. Moreover, dentists and gnathologists should consider both static and dynamic components of the patient’s occlusal scheme (see Table 5).

A static occlusal examination includes detecting teeth rotation, spacing, overjet and overbite (see Table 4) (including open-bites and cross-bites) that can reveal occlusal instability due to the presence of recurrently fracturing teeth and changes in tooth shape or position associated with indentations on lateral borders of the tongue and buccal mucosa related to bruxism tendency. In addition, dentists usually measure resting vertical dimension (RVD) and occlusal vertical dimension (OVD) through the Willis gauge, which is a tool that registers, in millimeters, the distance between the maxilla and mandibula. On the other hand, a dynamic occlusion examination refers to the study of teeth contact during mandibular movements, assessing the centric occlusion and intercuspal contacts marked up using articulating paper or a photographic record [51]. It should not be underestimated that physiological occlusion in adults can deviate in one or more occlusal parameters (Table 2) from the theoretically ideal one. Since this “well-adapted” occlusion is also aesthetically pleasing to the patient and has no pathological manifestations, it does not require any medical or orthodontic intervention. In fact, dentists should respect the biological variation in form and appearance of occlusion coherently with its function [52].

Actually, physical examination also includes palpation, following the direction of muscle fibers causing pain that can radiate into neighbor areas (periauricular, occiput, neck, shoulders) [53]. The palpation should address masticatory muscles (i.e., temporalis, superficial and deep masseter) and the surrounding neck and shoulder muscles that can highlight the location of pain and myogenous TMD. Notably, the temporalis muscle is divided into three portions (anterior, medial and posterior) that should be evaluated individually, as well as the digastric muscle during the opening movement, sub-occipital and sternocleidomastoid. Since lateral and medial pterygoids are not directly touchable, clinicians should therefore examine them using the resistance of hands during contractions. Interestingly, it has been hypothesized that medial pterygoid muscles could influence the opening pressure of the auditory tube, causing the “ear fullness” symptom in those patients with ear-related TMD. In detail, the dentist or therapist (both physiotherapist and osteopath) should bilaterally palpate masticatory muscles, placing one finger extra-orally and another one intra-orally, to detect hypertrophy, tenderness or pain, especially in muscle insertions. Moreover, postural evaluation should consider the upper cervical vertebral spine (C1, C2 and C3) and the cranial morphology of TMD patients since cervical dysfunctions could play a pivotal role in the development and maintenance of SD and TMD symptoms [54]. In fact, the TMJ degenerative process induces a backward-positioned jaw, which reduces pharyngeal airway capacity, altering the cervical posture in a compensatory forward head position for the decreased airway volume in the upright position [55]. Finally, clinicians should not overlook the psychological and stress status of the patients, since it can be involved in the etiology of some TMD, including bruxism [56]. When medical history and physical examination are equivocal, imaging instruments such as radiographic examinations (i.e., panoramic, planography and transcranial radiography), Computed Tomography (CT) and Magnetic Resonance Imaging (MRI) can be valid tools in the diagnostic path. TMJ radiographs are useful to collect information about morphological and anatomical characteristics between the condyle, articular tubercle and fossa. In detail, panoramic radiography is used to reveal osteophytes, fractures or other bone alterations, whereas planography is more accurate than panoramic radiography in spotting details in the styloid and mastoid process and the zygomatic arch. In the sagittal and coronal planes, the planography can also document the position of the condyle in relationship to the fossa during the MMO. Transcranial radiography provides a detailed evaluation imaging of the condyle, fossa and articular tubercle, with a large overlap in skull bones [57]. Notably, the most performed variation of CT in dentistry is the cone-beam (CBCT), which is useful to view skeletal and dental tissues involved in degenerative joint processes (osteoarthritis) using a low dose of radiation. MRI can instead confirm any disc-related TMD due to its ability to detect early abnormalities in the location and morphology of TMJ. Additionally, ultrasonography is less expensive than MRI and allows the diagnosis of an internal TMJ derangement [58,59]. However, these instruments are typically reserved for patients with persistent symptoms and for those in which conservative therapy has been ineffective.

## 6. Cranial–Temporomandibular and Stomatognathic Rehabilitation Approach

Multidisciplinary management with a focus on conservative and complementary therapies is currently recommended for patients who present TMD or orofacial pain. However, evidence about cranial–temporomandibular and stomatognathic rehabilitation (CTS-R) in patients affected by neurological disorders is still lacking (see Table 2).

Conservative and rehabilitative management should aim to decrease orofacial pain and muscle contractures/spasms, improving TMJ function, despite the variety of TMD and SD types that clinicians could find in the population of neurological patients. Notably, Zapata-Soria et al. [60] identified some CTS-R interventions in post-stroke patients, including therapeutic jaw exercises. It seems that both jaw opening and head lift exercises can improve digastric and mylohyoid muscle thickness as well as hyoid bone movements. In fact, the digastric muscle assists the depression and retrusion of the mandible during the following breathing, swallowing and chewing activity [26]. According to Oh et al. [27], the administration of postural alignment exercises can be a promising approach to restore neck mobility and TMJ opening function, especially in post-stroke patients. It is not surprising that a postural re-education approach can be effective in TMJ pain relief because of the strictly biomechanical relationship among stomatognathic skeletal elements, the cervical spine and the shoulder girdle [26]. In this context, postural exercises, such as head posture adjustments and the correction of the mandibular position and tongue [61], can be easily adapted and administered to a variety of neurological diseases.

**Figure 4:**
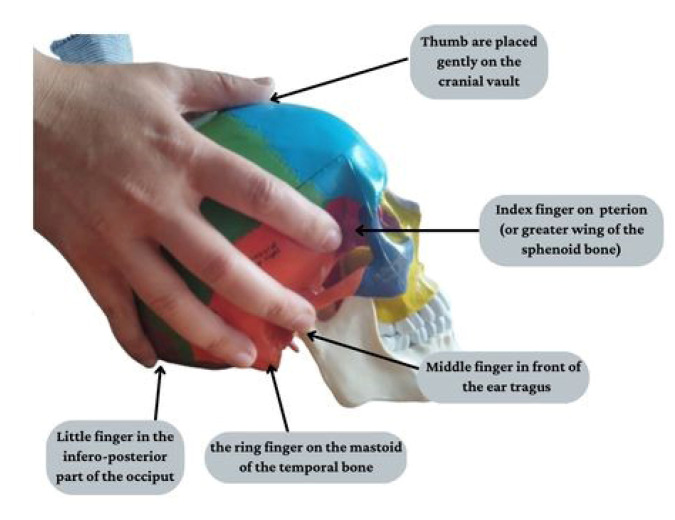
Illustration of how “Sutherland technique” is practically performed on a skeletal model.

Indeed, specific coordination exercises, including open-close or lateral mandibular movements [62], can be more useful in SCA and MS patients with cerebellum impairments since these exercises are effective to promote balanced and synchronized muscle activity, reducing muscle pain. Moreover, muscle strengthening exercises contribute to increasing the range of motion of the mandibula by the administration of isotonic jaw opening exercises with resistance, which inhibits jaw-closing muscles (i.e., masseter and temporalis), improving TMJ opening range and pain relief [63]. In this vein, it has been [64] suggested that exercise therapy, in addition to manual treatments, postural exercises and jaw mobilization, can be the most effective conservative and complementary management for orofacial pain and TMJ mobility. In particular, TMJ can be manipulated through myofascial release (MFR), in which the operator palpates muscles and soft tissues, producing a compression in tenderness points. Balanced ligamentous tension (BLT) comprises a series of techniques that provides both compression and passive approaches to place a joint in “balance” when moved in different planes. Among the manual treatments, cranial–sacral therapy (CST) consists of hands-on gentle manipulation of the skull and sacrum, which are bidirectionally linked through dural attachments [65]. Using this light pressure, the osteopath should release myofascial restrictions, identified through palpation, and restore mobility and reduce pain for patients [66]. Generally, the five-finger bilateral grip or “Sutherland’s technique” is a common means for the evaluation and treatment of cranial dysfunctions. The patient is supine and relaxed, while the osteopath is sitting behind him/her, with his/her hands bilaterally placed on the head of the patient. In detail, the fingers are placed in the following manner: (i) the index finger on the pterion, (ii) the middle finger in front of the ear’s tragus, (iii) the ring finger on the mastoid of the temporal bone, (iv) the little finger in the inferior-posterior part of the occiput, (v) while the thumb is placed gently on the cranial vault [67] (as shown in Figure 4).

Actually, the use of the occlusal splint to treat TMD is common in dentistry. In fact, these devices promote the correction of vertical dimension, TMJ realignment and repositioning, also providing cognitive awareness [68]. The mechanism of action consists of reducing electromyographic jaw muscle activity in the short term; however, long-term outcomes are still unclear due to the adaptive mechanisms of muscles [69].

Interestingly, Umay et al. [28] performed a combined protocol in post-stroke patients using intra-oral cold stimulation, strengthening oral exercises and intermittent galvanic stimulation to the masseter muscles for thirty minutes a day. The administration of functional electric stimulation prevented muscle atrophy, promoting compensatory mechanisms and coordination.

Another conservative and complementary approach that needs to be mentioned is the psychological management of pain, which should be included in the multimodal rehabilitation approach of TMD patients [70]. In fact, neurological disorders can cause psychological sequelae (i.e., anxiety and depression symptoms), which can exacerbate the TMD’s or SD’s symptoms. Breathing and relaxing exercises in which therapists guide the diaphragmatic inspiration, through hands on the rectus abdominis, are useful to promote muscle release and psychological wellness [71,72]. This is why counseling and behavioral approaches and relaxation techniques to manage pain could be adjunctive promising treatments, personalizing rehabilitation in a more centered-care way.

## 7. Discussion

As far as we know, this is one of the few reviews [11,60] that deal with SD and TMD and their rehabilitative approach in patients affected by neurological disorders. In fact, this issue is often overlooked by clinicians and therapists when it would need more attention, especially for the possible improvements in the most important functions of life: speech, eating and breathing, besides the postural alignment. It is noteworthy that evidence highlighted the correlation and the relationship between the neurological disorder and the onset of SD or TMD [14,22,36,37,38], while the literature about CTS-R intervention remains poor or limited to physical exercises for the post-stroke population [25,26,27,60] (see Table 2). Indeed, we have focused not only on the pathophysiological mechanism but also on the diagnostic work-up and treatment, pointing out the importance of a multidisciplinary and personalized approach.

Understanding the loading of SS and the existence of myofascial tension, articular dysfunctions and parafunctions such as bruxism are fundamental in delivering the most tailored functional evaluation and training in these patients. In fact, in neurological patients, it is not easy to address the right diagnosis due to patient compliance and the common overlapping between neurological disorders and SD or TMD. Physical examination should be meticulously performed, investigating patterns of occlusal contacts, mandibular opening movement and muscle tenderness. In this way, clinicians can collect indispensable information for planning primary and tempestive treatment.

Additionally, Botox injection and dry needling have been suggested to manage orofacial pain in myogenous TMD, especially when other strategies have already failed [73]. In particular, dry needling is less invasive than Botox injection, and it seems to be superior in reducing pain and improving the jaw range of motion, as confirmed by Kütük et al. [74]. Other kinds of medications, including corticosteroids, benzodiazepines and antidepressants, can also be used in TMD patients [75]. However, neurological patients have already taken many of these drugs for their pathology, and when ineffective, non-pharmacological approaches such as therapeutic exercises, manual techniques and physical therapy, as well as occlusal splints, should be considered as the primary intervention in these patients. Given that most medications induce muscle relaxation to reduce spasms and contractures, some authors [76] proposed the administration of relaxation exercises of masticatory muscles to improve TMD range of motion and reduce pain. In this context, static relaxation exercises can be more effective than standard active exercises.

Today, clinicians and therapists should consider the whole person as a unique and global system in which muscular and fascial components can influence body posture, SS and its functions (chewing, speaking and swallowing), which are almost impaired in neurological patients. Some authors stated [77,78] that the SS should be considered a part of the proprioceptive system, among balance, sight and postural control of the whole body. This could explain why manual treatments (such as OMT), physical exercise and postural re-education can be more effective than other pharmacological or non-conservative treatments. In fact, these treatments have been shown to act on the proprioceptive system and then sensorimotor integration, including the brainstem, subcortical and cortical centers, cervical region, proprioception and body posture. Given that, posture and gait training should be used with CTS-R since cervical, TMJ structures and lower muscles could influence each other through the fascial system; thus, it forms a single body system [79,80].

Consequentially, an altered pattern of movements in TMJ may cause an overload in masticatory muscles (i.e., posterior temporalis, ipsilateral external pterygoid and contralateral temporal anterior pterygoid, contralateral internal pterygoid). These alterations have repercussions on the upper trapezius and contralateral sternocleidomastoid, which determine flexion and side deviation of the head, also involving the ipsilateral shoulder (levator scapulae, omohyoid) and spine muscles (such as gran dorsi and iliopsoas) that have insertions on the lumbar tract and ileum. In this way, the cranial–mandibular structures could influence lower body extremity, not only in static posture but also during gait [11,81]. However, the relationship between human posture and TMD remains one of the unsolved research questions. Future studies should consider the chance for conservative and complementary approaches to induce appropriate neuroplastic changes, integrating them with neurologic exams, monitoring of body balance and coordination control systems.

Furthermore, a multidisciplinary approach is strongly recommended, as patients can benefit from complementary therapies, including OMT, posture re-education, physical exercises and occlusal splint therapy, for SD and TMD. Finally, a co-work among gnathologists (or even dentists), physiotherapists, osteopaths and neurologists is extremely important in achieving better outcomes and avoiding unnecessary treatments.

Since a standardized protocol for the evaluation and treatment of SD/TMD in neurological disorders is still lacking, herein, we have reported some evidence-based clinical advice about the administration of multidisciplinary conservative approaches for neurological patients who have SD/TMD:

- Parafunctional activities, such as bruxism and day clenching, have been found in PD and MS patients [11,19,22,23], causing muscle pain and harmful effects on tooth enamel. Currently, there are no treatment methods to make these alterations stop. Occlusal splint therapy can reduce bruxism and clenching symptoms, acting on a negative feedback mechanism that greatly decreases muscle activity, maintaining a normal activation threshold for the muscle protective reflex. In addition, MT and MFR could be useful to induce masseter, temporalis and neck muscle relaxation that are associated with these symptoms [65];
- Reduction in TMJ movements due to painful muscle contractions is a common TMD feature in neurological conditions, especially in OMD, PD and SCA [21,24,29,35,37,38]. However, evidence-based treatments have been documented only for OMD subjects, suggesting the use of Botox injections in specific head and neck muscles, such as platysma, lateral pterygoid and temporalis, to reduce muscle spasms [38]. Coordination exercises of TMJ through opening and closing the mouth, using a mirror or fingers bilaterally, to promote symmetrical movements may also be of help [62,82,83]. Additionally, MT and/or OMT could induce muscle release and restore pain thanks to the discharge of endogenous opioids due to therapeutic touch [9,65];
- Muscle weakness and reduced TMJ functions were found in post-stroke survivors as a result of the acute onset of the brain damage [25,26,27,28]. In this clinical condition, authors [26,27,60] suggested the administration of specific isometric and isotonic exercises to improve jaw muscle strength and postural programs [61,84], including exercises to increase TMJ and neck mobility. To restore oral muscle strength, some evidence supported the administration of specific exercises for labial, intrinsic tongue and masticatory muscles, which can be helpful in managing dysphagic symptoms [28].

## 8. Conclusions

To summarize, although SD and TMD in neurological patients do not seem to be uncommon, a standard diagnostic or rehabilitation approach is still lacking. Clinicians and therapists should consider the role of SS and TMJ structures during functional training, given their fundamental role in swallowing, chewing, breathing and speaking. To better manage SD/TMD, neurological patients should be seen as a single unit, in which the stomatognathic complex works together with the cervical spine and lower extremity in maintaining body posture and spinal alignment. Future studies are needed to fill this existing gap in the individuation and treatment of SD and/or TMD in the neurorehabilitation field.

## Figures and Tables

**Table 1:** Search strategy used for the attrition of studies.

Neurological Disorders	Temporomandibular/Stomatognathic Diseases	Conservative and Rehabilitative Approaches
“Parkinson’s disease” OR “Multiple sclerosis” OR “Spino-cerebellar ataxia” OR “stroke” OR “oro-mandibular dystonia” OR “movement disorders”	“Temporomandibular joint disorders” OR “temporomandibular joint dysfunctions” OR “stomatognathic disease” OR “bruxism” OR “disc displacement” OR “temporomandibular myofascial pain” OR “orofacial pain”	“Physical exercise therapy” OR “manual therapy” OR “osteopathic manipulative treatment” OR “cranial-sacral therapy” OR “physical therapy” OR “occlusal splint therapy”

**Table 2:** Description of studies that dealt with SD/TMD in patients with neurological disorders.

Reference Number	Association between SD/TMD and Neurological Disorder (Yes or No)	Stomatognathic Disease	Diagnostic Tools	Musculoskeletal Structures Involved	Conservative and Complementary Treatments	Major Findings
Multiple sclerosis (MS)						
Costa et al. [19]	Yes	TMD, bruxism, tooth hypersensitivity and hyposalivation	Clinical intra- and extra-oral examination	Suboccipital and cervical muscles	Endodontic intervention, occlusal adjustment and behavioral education	The endodontic treatment met the aesthetic pleasing of the patient
Williams et al. [20]	Yes	Jaw clenching/bruxism	Ultrasonic pulsed phase-locked loop (PPLL) and change in acoustic pathlength (∆L) as the measure of intracranial distance	Masticatory muscles, temporal bones and TMJ	NA	Jaw clenching/bruxism was associated with the displacement of the temporal bones and expansion of the cranial cavity in MS patients compared to healthy control
Spinocerebellar ataxia (SCA)						
Ferreira et al. [21]	Yes	Increased masticatory muscle activity and reduction of maximal molar bite force	RDCTMD, electromyographic activity, muscle thickness and maximum bite force	TMJ structures, masseter and temporalis	NA	SCA is characterized by functional and electromyographic alterations in SS, especially in chewing and bite force
Parkinson’s disease (PD)						
Choi et al. [22]	Yes	Jaw tremor, bruxism amd TMJ rigidity	Diagnosis of TMD was considered using ICD-10 code K07.6	Masticatory muscles, TMJ structures and cervical spine	NA	The authors stated that PD patients have a high risk to develop TMD; conversely, individuals affected by TMD have more risk to develop PD in the future
Verhoeff et al. [23]	Yes	Bruxism (both sleep and awake bruxism), TMD and orofacial pain	The authors created an 18-item questionnaire, reporting: (i) chronic pain; (ii) the DC/TMD; (iii) oral behavior; (iv) DC/TMD symptom questionnaire; (v) TMD pain screener	Masticatory muscles, TMJ structures and cervical spine	NA	There are correlations between PD and bruxism, and PD with TMJ pain
Oromandibular dystonia (OMD)						
Handa et al. [24]	Yes	Myofascial pain in masticatory muscles and dental problems	Differential diagnosis between OMD and TMD by clinical examination and ICD-10	Masticatory muscles, TMJ structures and dental arches	NA	Since OMD shares clinical features with TMD, they are often misdiagnosed with the risk to receive unnecessary treatments
Stroke						
Alvater Ramos et al. [25]	Yes	Disc displacement and myogenous TMD	TMD diagnosis was performed using RDCTMD; physical mechanical pressure on trigger points was tested using the algometer Wagner PAIN TES and Pressure Pain Threshold Test, while cervical ROM was assessed using a Sanny Fleximeter	Cervical lateral-flexors muscles, TMJ and masticatory muscles	NA	The authors found that post-stroke patients manifested augmented muscle tone and reduced cervical ROM on the affected side, suggesting that the musculoskeletal alterations caused by a stroke can predispose to TMD
Choi et al. [26]	Yes	Dysphagia	Dysphagia was confirmed by a video-fluoroscopic swallowing study	Suprahyoid muscles (digastric and mylohyoid muscles) and hyoid bone movements	Jaw opening exercise (isometric and isotonic) and head lift exercise	JOE and HLE were useful to improve supra-hyoid muscle strength and thickness. However, JOE required less effort than HLF
Oh et al. [27]	Yes	Decreased TMJ function	Clinical examination with craniomandibular index and limited range in opening mouth; swallowing function was assessed using MASA	TMJ structures, masticatory muscles, neck and shoulder muscles	Stomatognathic alignment exercise program (exercises to increase the mobility of the neck and TMJ), head and neck posture exercises and anterior chest stretching exercise)	Stomatognathic alignment exercises were useful to improve TMJ and swallowing functions
Umay et al. [28]	Yes	Swallowing dysfunction, masticatory and swallowing muscles weakness	Swallowing intervals and motor action potentials (MAPs) of trigeminal, facial and hypoglossal nerves were measured	Swallowing muscles, masticatory muscles, hyoid bone and neck structures	Thermal stimulation (to radix of tongue, palate, tonsillar plica, and oral mucosa); oral motor strength exercises for labial, intrinsic tongue and masticatory muscles; intermittent galvanic stimulation	After four weeks of treatment, significant recovery in swallowing, motor and general functional levels of the patients was provided

Legend: SD (stomatognathic disease), TMD (temporomandibular disorder), TMJ (temporomandibular joint), RDCTMD (Research Diagnostic Criteria for Temporomandibular Disorders), JOE (jaw opening exercise), HLF (head lift exercise), MASA (Mann assessment of swallowing ability).

**Table 3:** Risk of bias assessment of randomized trials with RoB 2.

Reference	Randomization Process	Effect of Assignment on Intervention	Effect of Adhering to Intervention	Missing Outcome Data	Measurement of the Outcome	Selection of the Reported Results
Choi et al. [26]	SC	SC	L	SC	SC	SC
Oh et al. [27]	L	L	L	L	SC	SC
Umay et al. [28]	L	L	L	L	L	L

Legend: L (low); H (high); SC (some concerns).

**Table 4:** Risk of bias assessment of cross-section and case-control studies through Newcastle–Ottawa Scale (NOS).

Reference	NOS
Williams et al. [20]	4
Ferreira et al. [21]	4
Choi et al. [22]	6
Verhoeff et al. [23]	7
Handa et al. [24]	5
Alvater Ramos [25]	3

**Table 5:** Description of the main occlusal physiological parameters that dentists can measure during physical examination.

Main Occlusal Physiological Parameters	Description
Centric occlusion	Consists of a full occlusal contact between upper and lower teeth in habitual occlusion.
Incisal guidance	Consists of the influence of the contacting surfaces of the mandibular and maxillary anterior teeth on mandibular movements.
Canine guidance	Vertical displacement of the mandible due to gliding contact of the canine teeth, preventing potential damages.
Overjet	Defined as the horizontal overlap of the incisors, which can be augmented in the second occlusion class or reduced in the third class.
Overbite	Defined as the vertical distance between the incisal margins of the upper incisors and the incisal margins of the lower incisors. It can be increased in case of a deep bite or reduced in an open bite.
Occlusal vertical dimension (OVD)	Also known as the vertical dimension of occlusion and indicates the occlusion position of teeth in maximum intercuspation. A common trick is to ask the patient to say the word “Emma”, and after completing the word, the clinician has an estimate of OVD.
Resting vertical dimension (RVD)	Refers to a resting position of the mandibula. It happens when the maxillary and mandibular arches are not in contact with each other.
Freeway space	Defined as the neutral position attained by the mandibula as it is involuntarily suspended by the reciprocal coordination of masticatory muscles, with the maxillary and mandibular teeth separated.

## Data Availability

Not applicable.

## Abbreviations

SS	Stomatognathic system
TMJ	Temporomandibular joint
TMD	Temporomandibular dysfunction
SD	Stomatognathic disease
PT	Physiotherapy
MT	Manual therapy
OMT	Osteopathic manipulative treatment
CPG	Central pattern generator
MS	Multiple sclerosis
SCA	Spinocerebellar ataxia
PD	Parkinson’s disease
OMD	Oromandibular dystonia
RoB	Revised Cochrane risk of bias
NOS	Newcastle–Ottawa Scale
MPQ	McGill Pain Questionnaire
SF-MPQ	Short form McGill Pain Questionnaire
PPI	Present pain intensity
VAS	Visual analogical scale
VRS	Verbal rating scale
NRS	Numerical rating scale
FPS	Faces pain scale
HCDI	Helkimo Clinical Dysfunction Index
OVD	Occlusal vertical dimension
RVD	Resting vertical dimension
MMO	Maximum mouth opening
LEP	Laser-evoked potential
CT	Computed tomography
MRI	Magnetic Resonance Imaging
CBCT	Cone-beam computed tomography
CTS-R	Cranial–temporomandibular and stomatognathic rehabilitation
MFR	Myofascial release
CST	Cranial–sacral therapy
BLT	Balanced ligamentous tension
